# Gender and Age Differences in Preferences on Game Elements and Platforms

**DOI:** 10.3390/s22093567

**Published:** 2022-05-07

**Authors:** Carina S. González-González, Pedro A. Toledo-Delgado, Vanesa Muñoz-Cruz, Joan Arnedo-Moreno

**Affiliations:** 1 ITED Research Group, Department of Computer Science and Engineering, Universidad de La Laguna, 38204 Tenerife, Spain; petode@ull.edu.es (P.A.T.-D.); vmunoz@ull.edu.es (V.M.-C.); 2 Instituto Universitario de Estudios de las Mujeres, Universidad de La Laguna, 38204 Tenerife, Spain; 3 Estudis d’Informàtica, Multimèdia i Telecomunicació, Open University of Catalonia, 08018 Barcelona, Spain; jarnedo@uoc.edu

**Keywords:** online games, player preferences, gameful design, gender differences, age differences, devices, platforms

## Abstract

This paper analyzes different player type models and game elements in the literature, particularly focusing on the case of online games. Research based on an exploratory study is presented; it aims to explore the different types of interaction with gameful digital applications. The study is based on a survey and provides findings from the literature review and empirical insights about users’ differences and preferences regarding game elements. The results reveal demographics regarding player profiles and the relationships between gender, age, culture, and the influence of different game design elements and platforms. The main contribution of this study fulfills the need for knowledge about the relationship between game element design, platforms/devices, and players (types and preferences).

## 1. Introduction

Personalizing gameful systems to each user is important because personalized interactive systems are more effective than one-size-fits-all approaches [1,2]. It requires a dynamic adaptation to the user’s behaviors in response to any situation. This approach offers users system-tailored content and services, developing content and functionality for each need based on the user’s characteristics [3]. To create actual long-term behavior change, the entire gameful system should be designed to meet the needs of each user; in consequence, early long-term studies are being carried out to investigate this topic further (e.g., [4,5,6]).

Some basic elements must be taken into account before designing a personalized gameful experience: defining the user profile, the content, and the functionality, as well the interface elements. Researchers have been conducting initial studies regarding a diverse set of dimensions for personalization, such as personality [7], gender [8], persuadability [9], and player types and design elements [10]. Nevertheless, how gameful interactions can be personalized and which factors can be used in online gaming are still largely unexplored [11].

Therefore, despite the preliminary findings of these works, this scope has not been explored in depth yet. There is an open research niche regarding relationships between users and their preferences when interacting with gameful environments (in which specific game design elements are used) beyond a primary player taxonomy. To fill this gap, the objective of the present study is to gather preliminary information that will help deepen the personalization of gameful design elements in online gaming.

We can find many types of online games with different characteristics. Some of the most common online games are massively multiplayer online (MMO), board, real-time strategy (RTS), simulation, first-person shooter, action and adventure, arcade, sports, puzzle, and casino games. The massively multiplayer online (MMO) games are among the most popular online games. Players use a network and interact with other players from all over the world in the virtual game room; titles include World of Warcraft, Star Wars The Old Republic, Dota, Guild Wars, and Black Desert Online. Online board games are animated versions of traditional board games such as Monopoly, Carcassonne, Catan, and Scrabble.

On the other hand, real-time strategy (RTS) or strategy games involve strategies to play and win the game, but RTS games move in real time, and players can play at once without taking turns. Examples include Starcraft, Age of Empires, Clash of Clans, and Total War. We have other types of games, such as simulation games, that involve taking control of aircraft (FlyWings, Flight Simulator), ships (Ship Simulator Professional), vehicles (Train Simulator, Euro Truck Simulator, DIRT Rally, etc.). In these games, players can learn how to control these vehicles, and the games can be used by professionals; for example, pilots can be trained using airplane simulators. In first-person shooter games, the player is the protagonist, and the game is viewed through the player’s eyes. These kinds of online games are trendy. Examples include Counter-Strike, Battlefield, Halo, Quake, and Battlefront. Other types of games are action and adventure games, which generally start with a story; players know the mission and have to figure out how to complete it.

Furthermore, games can use puzzles to advance levels. Most of these adventure games are rich in animation with a strong storyline. Online action games also include fighting games, space adventure games, etc. Players have to achieve some objectives (StarBound, Path of Exile, Terraria, Castle Crashers). Another very popular type of game is the arcade games, which are very popular among all ages of people. Some popular online arcade games are Pac-Man, Space Invaders, Asteroids, and Pong. In sports games, users can play real-world sports such as soccer, basketball, and F1. The most popular sports games are usually based around specific popular sporting events. Plus, one can compete against another player, team, or the computer itself. Examples include Football Manager, F1, and NBA 2K. Puzzle games usually are brain games with no action involved for players that love to solve challenging puzzles: Mahjong, Bubble Town, and Candyland, among others. Other online games that are gaining high popularity are casino games. These replicate the games available in real casinos, involving real money transactions with real bonuses and prizes (Wheel of Fortune, Super Slots, European Roulette, American Blackjack).

This work will focus on online games’ game elements (components, mechanics, and dynamics), typically characterized by simple rules and reduced demands on time and learned skill, in contrast to more complex hardcore games. Most of them can be played on web browsers, mobile phones, and tablets. Furthermore, most casual games have similar basic features of simple gameplay such as being a puzzle game, having a straightforward interface using a one-button mouse or smartphone keypad, being based on card or board games, and usually having continuous play with no need to save the game.

Accordingly, we set the following research questions to be answered through the development of the present exploratory study:

RQ1 : Are there gender differences in preferences on gameful elements and platforms/devices in online gaming?
RQ2 : Are there age differences in preferences on gameful elements and platforms/devices in online gaming?

Besides answering the proposed research questions, this exploratory work looks forward to contributing to human–computer interaction (HCI) research by suggesting new proposals for personalized user interfaces for online games.

This paper is organized as follows: First, the theoretical background on personalization, models of player types, and game design elements is described. Then, related studies about the relationship between player types, preferences, and game design elements are presented. Then, the study and methods, with their results, are described. Finally, the main findings and conclusions are analyzed.

## 2. Background

This section presents the background related to personalization in games, models of player types, and game design elements.

### 2.1. Personalization and Games

Personalization and customization are concepts commonly used interchangeably in the literature; however, they should not be blended together. While personalization is the degree to which the system tailors the content to individual tastes (system-tailored), customization refers to the user deliberately tailoring content by choosing options and/or creating new content (user-tailored) [12]. Regarding technological environments, some authors refer to personalization as the specification of the desired web layout and content that matches the user’s interests and preferences, together with tools and options that employ mechanisms to offer content and layout for each individual user [13].

Personalization is a term initially coined by marketing professionals during the past century. The original idea was tailoring an offering to better suit a certain customer group through segmentation [14], which is considered the basis of a good marketing strategy. Through segmentation, campaign design was more effective in identifying users and selecting a good target market. Personalization provides value to the customers of a company, and customization enables users to explore different possibilities in their products or services. Therefore, it is a highly relevant topic in marketing and segmentation. The definition of user “personas” is expected to define the ideal profile of a potential buyer or user in marketing and sales. A persona definition clarifies these characteristics, behaviors, and relevant needs of the target users [15].

Accordingly, personality, conceived as an inner tendency or predisposition for a person to act in a certain way, is a relevant topic that should be studied to help understand users of interactive technologies from the perspective of motivation, i.e., how users interact with the system or how they can be segmented according to their behavior. In this regard, Myers [16] provides the Myers–Briggs Type Indicator (MBTI) based on eight scales (Extraversion vs. Introversion, Sensing vs. Intuition, Thinking vs. Feeling, and Judging vs. Perceiving), where an individual will be paired with four of them. It was considered a useful personality scale years ago, designed to help a person identify some of their most important personal preferences. A player satisfaction model, BrainHex [17], provides a comparison between MBTI and diverse playing style preferences. This model presents seven player archetypes: Seeker, Survivor, Daredevil, Mastermind, Conqueror, Socializer, and Achiever [18].

In recent years, the five-factor model (FFM) of personality, commonly known as the Big Five [19], posits that the perception of personality is formed by five broad factors or dimensions of personality: factor O (openness or openness to new experiences), factor C (conscientiousness or liability), factor E (extraversion or extroversion), factor A (agreeableness or kindness), and factor N (neuroticism or emotional instability). The recent literature agrees that the FFM model is a more accurate representation of an individual’s personality than the MBTI model; therefore, it is a preferred representation for understanding potential personalization factors.

Deepening the perspective of user modeling as the base of any type of personalization, researchers have made a great effort to “simplify” this complex labor by creating player taxonomies. This allows researchers and designers to work with models that can be easy to handle within the complexity and individuality of each user. However, Hamari and Tuunanen [20] suggest that the topic of player typologies (how players play or how they can be segmented according to their behavior) has not been exhaustively studied yet. They reviewed the existing player type models and synthesized their commonalities into five key dimensions of player motivations: Achievement, Exploration, Sociability, Domination, and Immersion.

These dimensions broadly fit with the most used taxonomy in gameful design literature, Bartle’s Player Types [21] (Player, Socializers, Killers, and Achievers), an observation that is corroborated by a review on gamification design frameworks [22]. However, it was explicitly created for multiuser dungeons (MUDs), and it should not be generalized to a gameful design. Kim [23] argues that, in practice, the four types defined by Bartle do not work in the case of social and casual gaming. She developed an alternative taxonomy about playing styles based on Bartle’s model, showing new motivational patterns focusing exclusively on casual games and social gender. At the same time, Yee [24] proposed a set of elements that complement Bartle’s model on the basis that player types could be highly correlated with each other. Therefore it would be challenging to use Bartle’s model practically. He updated the model considering the following dimensions: achievement (advancement, mechanics, and competition), social (socializing, relationship, teamwork), and immersion (discovery, role-playing, customization, and escapism). However, demographic issues have not been considered. There are several works (e.g., [25,26]) that have assessed players’ typologies relying on demographic factors (e.g., age, gender, education).

Another model is the Hexad User Types [16], which defines the following player types:

- Socializers: They are motivated by relatedness. They want to interact with others and create social connections.
- Free Spirits: They are motivated by autonomy and self-expression. They want to create and explore.
- Achievers: They are motivated by mastery. They are looking to learn new things and improve themselves. They want challenges to be overcome.
- Philanthropists: They are motivated by purpose and meaning. This group is altruistic and enjoys giving to other people and enriching the lives of others in some way with no expectation of reward.
- Players: They are motivated by rewards. They will do what is needed to collect rewards from a system.
- Disruptors: They are motivated by change. They want to disrupt the system, either directly or through other users, to force positive or negative change.

Some authors [1] suggested a table of game design elements for each user type using correlation analysis of the Hexad User Types with different game design elements. Their findings demonstrated the usefulness of the Hexad model as a measure of preferred design elements (based on a list of suitable gameful design elements for each one of the player types proposed by Marczewski [27]).

In the following subsection, we describe different game design elements and their relationship with some player types, such as those defined in the Hexad User Types model.

### 2.2. Game Design Elements

There are different game design elements [28,29,30], and some authors suggested other relationships between user types and gamification designs, game element preferences, and mechanics [31,32,33]. For example, we present six elements related to the user types proposed in the Hexad User Types: leaderboards (i.e., Players), teams (i.e., Socializers), challenges (i.e., Achievers), the voting mechanism (i.e., Disruptors), gifting (i.e., Philanthropists), and exploration (i.e., Free Spirits) [34]. In the following, we describe each one of these game design elements:

- Leaderboard: A leaderboard (a suggested element for Players) is a board for displaying the ranking in a competitive environment. Many minor design decisions are involved in implementing leaderboards that may influence their impact. Given the multiple ways that leaderboards can be presented and the increasing number of non-game applications that rely on them, a better understanding of the psychological implications of being placed in a particular leaderboard position is needed.
- Teams: A team (a suggested element for Socializers) is a structure that involves two or more players working together towards a shared objective. More precisely, a team is described by two or more individuals who socially interact; possess one or more common goals; are brought together to perform organizationally relevant tasks; exhibit interdependencies concerning workflow, goals, and outcomes; have different roles and responsibilities; and are together embedded in an encompassing organization.
- Challenges: A challenge (a suggested element for Achievers) is an activity that needs great effort to be completed successfully and therefore tests a person’s ability. The idea is to ensure there is always a challenge for players to take. It provides users with a sense of autonomy by choosing which challenges to pursue, which may be enjoyable to Free Spirits, in contrast to challenges that must be completed in a limited amount of time.
- Voting Mechanisms: A voting mechanism (a suggested element for Disruptors) is a method by which users select between different choices. This mechanism can be designed from diverse perspectives, such as one-to-one, one-to-many, many-to-one, and many-to-many, from the more restrictive approach to the most permissive; positive/negative voting (up/down); and individual/collaborative.
- Gifting: A gift (a suggested element for Philanthropists) is an action that allows people to give or share items with other people to help them achieve their goals. This action is proposed as easily transferable virtual items or karma points, possibly a motivating strategy for Philanthropists and Players, who aim to help others with items gained in the form of rewards, or it could be enjoyable for Socializers.
- Exploration: Exploration (a suggested element for Free Spirits) is the freedom to try different things in the system and accomplish tasks in other ways, which are not mandatory to play but can be re-entered any time later.

The primary game dynamics involved in casual games are reward, status, achievement, and competition. Mechanics associated with its main game elements are Leaderboards/Competition, Challenges/Achievement, Points/Credits/Rewards, Levels/Status, Voting, and Exploration [20] (Table 1).

The following section describes different studies about user preferences and their relationship with game design elements.

### 2.3. Related Works

Different studies of user preferences are related to diverse game design elements [35,36]. Ferro et al. [37] studied the relationship between player types and personality traits in gamified systems, aiming to identify potential relationships with game elements. This connection (based on their knowledge and experience) provides a dynamic toolbox for an adequate design of gamified systems, specifically targeting users in an intrinsically engaging and motivating way. However, they propose a purely theoretical model. Likewise, Xu [38] links game mechanics (a set of rules and feedback loops intended to produce enjoyable gameplay) to player types based on Bartle’s model [20].

Another study conducted by Gil et al. [39] demonstrated that each person has their own personality and tastes. Therefore, certain game design elements can motivate them and may be irrelevant or non-engaging for other people. Their work empirically validated the effectiveness of diverse game design elements and the adequacy of such elements for player types. They employed a personality-based questionnaire to infer the users’ player types (a modified version of the player type questionnaire proposed by Marczewski [27]) and diverse implementations of game design mechanics to determine the motivation for each of the user types. In the survey proposed by Marczewski to find primary user types, researchers asked participants to freely choose the assignments to solve by performing actions based on game design elements.

Jia et al. [40] studied the relationships between an individual’s personality traits and perceived preferences for various motivational affordances used in gamification. They considered that most gameful applications use multiple combinations of motivational affordances (game design elements) but that these combinations are not designed for a specific use. The study was focused on personality traits by using a derivative version of the “Big Five” model [18]. Initially, participants were asked to complete an assessment test of the Big Five factors of personality. Next, they were asked about their perceptions of 10 motivational affordances (game design elements) using demonstrative videos. As a result, through a correlation study, the authors linked motivational affordances with different game design elements.

Differing from these empirical studies, many related proposals in the literature do not seem to be validated and are based only on assumptions or the researcher’s experience. Accordingly, Xu [32] presents a summary table aimed at helping guide designers and managers in using the appropriate game design elements to engage different types of users. It has been designed to highlight how different player types may respond to some of the most popular design elements. A comprehensive list of elements is compiled and linked to Bartle’s Player Types. However, it seems that it is not based on solid evidence.

In this way, Orji et al. [41] present a personalized approach that will best motivate a particular type of gamer by mapping common game elements and mechanics; in addition, Ferro et al. [37] proposed a table to identify player types, personality traits/types, and game elements and mechanics.

Yee (2006) [24] presents an empirical model of player motivations in online games. The model consists of 10 motivational components (advancement, mechanics, competition, socializing, relationship, teamwork, discovery, role-playing, customization, and escapism) that can be grouped under three umbrella categories (achievement, social, and immersion components) (Table 2).

Current studies can be synthesized into five key dimensions regarding the motivations of play/orientation of the player: Achievement, Exploration, Sociability, Domination, and Immersion [20]. Although the motivational factors are not precisely player types, they can be seen as a possible basis for psychographic segmentation based on motivations for play [42,43].

In the next section, we describe an exploratory study about player types and their preferences for game elements [44].

## 3. Study Design and Methods

The present study is based on an online survey, which allowed us to collect data from a wide range of participants worldwide. We designed the survey through an iterative process by involving various actors in the following steps:

(a) Review: We reviewed the literature about player user types and game design elements.
(b) Design: We created a preliminary suggestion of statements about how participants enjoy different ways of implementing each game design element based on the findings from the literature review and then reviewed it with experts in human–computer interaction and game research.
(c) Ethical approval: The survey received clearance from the ethics committees of the institutions involved in this work.
(d) Translation: We translated all the statements and descriptions to each language version of the survey (from Spanish to English, Portuguese, and Catalan).
(e) Pilot test: We carried out a pilot survey with a sample of participants (excluding researchers or experts in the field.
(f) Activation and dissemination: We activated the survey and disseminated it via social media and nets.

The survey was enabled in an online service (using the LimeSurvey software). Participants were asked to complete a 15 min survey consisting of questions focused on their preferences while using gameful systems within digital applications, composed of five sections with 62 queries. The study dimensions were: the demography of the sample (age, gender, country, native language), gaming habits, interactions with gameful design applications, and different ways of implementing six game design elements.

Participants were mainly recruited through snowball sampling with the use of e-mails (both academic and non-academic environments), as well as through social networks (Facebook, LinkedIn, Twitter, and Reddit), game events, and learning management systems (LMSs) from the participating institutions. The total number of participants who answered the survey was 925. Note that participants were required to be at least 18 years old. The following section presents the main results of the exploratory study.

## 4. Results

Firstly, some of the main figures extracted from the survey results are presented. The analysis focused on three different perspectives is included afterward. These dimensions are the relation of gender with the player preferences, the link of age with preferences, and the preferences on game design elements. Table 3 shows that 815 participants filled in their gender, 41.47% females.

**Figure 1:**
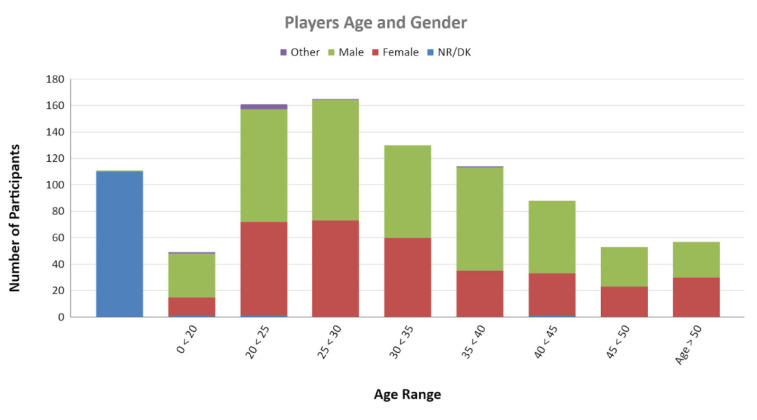
Age and gender of participants in the sample. Source: own elaboration.

The age distribution of the participants can be observed in Figure 1. The age interval containing the most participants is from 25 to 30 years. The number of participants decays smoothly, and above 75 years, we did not register any answers.

The geographical origin of the participants is diverse, as is their native language, as can be observed in Table 4. The most significant contribution to the survey comes from Spanish-speaking participants, followed by English speakers.

To answer RQ1 (Are there gender differences in preferences on gameful elements in online gaming?), we analyzed several data about gender differences and preferences.

The first dimension analyzed is related to the number of days of the week the speaker plays a significant amount of time. Table 5 shows the statistically significant difference regarding gender in the aggregated number of days in which respondents play, increasing from approximately 3 to 3.8. Table 5 displays that females only represent 36.14% of the time the participants play games—the columns “Avg. Female”, “Avg Male”, and “Avg Other” represent the average number of days per week they play games.

As a consequence of this difference, to analyze the survey results from the gender perspective, the aggregated numbers of days are used to normalize the results, avoiding the bias induced by an uneven population in terms of game use.

The following research question focuses on analyzing if there are any gender differences regarding the type of device the participants prefer to use to play. Six different device types have been measured.

Table 6 shows aggregated values of the answers to the questions mentioned above. To notice if there is a difference from what is expected from the uneven population dedication, two additional columns have been calculated. The first one is “Female %”, which calculates the percentage of participants who use the device who are females. The column “Dif.” shows how much that percentage deviated from the expected value given the population distribution. A positive percentage indicates an overrepresentation of female preference for the device; on the contrary, a negative percentage indicates an underrepresentation of female preference for the device.

In Table 6, we can observe important differences related to gender and the preferred device. The most significant difference is found in the use of console (Female: −14.86/Male: 14.51), followed by hand console (Female: −9.55/Male: 7.08) and PC/Mac (Female: −7.37/Male: 7.51), showing less use of these types of platforms in women. In the case of male players, the difference shows less use of board games/card games (Female: 8.22/Male: −8.32), tablets (Female: 7.26/Male: −6.64), and smartphones (Female: −5.37/Male: 4.84). Despite the differences found, in the case of tablets, a low difference in the use of this device is observed.

We also asked about the participants’ experience with different elements. The questions were the following:

- QE1: Have you ever played a game that uses leaderboards (scoreboards on which the names, etc., of the leading competitors, are displayed)?
- QE2: Have you ever played a game that uses teams (numbers of persons associated in some joint action)?
- QE3: Have you ever played a game that uses challenges (explicitly tricky or demanding tasks, especially those seen as a test of one’s abilities or character)?
- QE4: Have you ever played a game that uses a voting mechanism (the action of giving a vote: to exercise the right of suffrage to express a choice or preference by ballot or other approved means)?
- QE5: Have you ever played a game that uses gifting (to endow or furnish with gifts)?
- QE6: Have you ever played a game that allows exploration (tasks built for the purpose of exploration, especially those constructed or selected for exploration or observation of the surrounding area)?

Table 7 shows the results of this analysis. It has the same structure previously described for Table 6. In this case, the most positive difference between genders is obtained for the use of gifting, although the difference does not seem to be significant.

The next dimension to be analyzed in RQ2 is the differences in the age of the participants. In Table 8, the aggregated number of days in which participants play games can be found. Additionally, it includes the percentage this number represents concerning the total and the difference from the expected percentage regarding the participant age distribution. As expected, the participants aged between 20 and 30 overrepresent the number of days played by the population.

Regarding the use of different platforms/devices, the aggregated numbers are included in Table 9. Nevertheless, to extract conclusions from the data, it is necessary to observe the percentage this number represents and the observed difference from the baseline distribution shown in Table 10.

Analyzing the behavior of the respondents, we can observe that age has a significant influence on the type of gaming platforms users prefer. Some technological supports are preferred at younger ages. That is the case of PC/Mac and hand consoles, the use of which is overrepresented in the answer by users in the range from 20 to 25 years. The use of hand consoles is especially relevant with a 9.27% overrepresentation of users of these young ages. On the other hand, other technologies are marginally more used by older users. That is the case with board/card games, tablets, and smartphones. The results are remarkable for the case of tablets, for which the use is 9.98% overrepresented in users of 40 < 50 years. Finally, the case of consoles is different from both cases mentioned above. In this case, middle-aged users between 30 and 35 make the most intensive use, with a 7.65% overrepresentation.

Regarding which player preferences exist on gameful elements in online gaming, we analyzed the user preferences. The results of how the elements have been distributed regarding the age of the participants are shown in Table 11.

The results show that the differences among age intervals seem to be much smaller, not significant for most analyzed elements. Nevertheless, it should be stated how the use of challenges is the only element that results in noticeable deviations regarding the age interval.

Using the results shown in Table 11, deviations are calculated in Table 12.

Analyzing the deviations in Table 12, it can be observed that, in general, the behavior is close to the participants’ age distribution. There are only a few cases where the percentage difference is more significant than 2% from the distribution of the age of the participants. That is the case of the use of leaderboards (QE1) in ages in the interval 40 < 45 (+2.01%), and more significantly in the case of using voting mechanisms (QE4) for all age ranges. Younger players overrepresent the use of these mechanisms (up to +6.34% for the range <20) while the older players are underrepresented (down to −4.19 for the range 40 < 45).

## 5. Discussion

Related to RQ1 about gender differences and preferences on gameful elements in online gaming, we can state that males dedicated more time to playing (63.16%) online games than females or other players. We observed gender differences regarding the type of device/platform the participants preferred to use to play; females presented the most significant preference for board games/card games, while consoles represented their least favored device. Tablets seem to be much more evenly used by both genders. In Table 6, we can observe critical differences related to gender and the preferred device/platform. Linked to game elements, the most positive difference between genders is obtained for the use of gifting, although the difference does not seem to be significant. The most crucial negative difference appears related to voting mechanisms, which have been recognized 12.33% less by females while playing. Overall, it can be observed how females reported less than expected number of game elements they have identified in the games they play compared to male participants. Further analysis is necessary to determine the causes of this difference.

Regarding the differences by age (RQ2), it can be concluded that age significantly influences the type of gaming platforms users prefer. However, the participants’ behavior toward their selected platform/device cannot be considered homogeneous. While participants of all ages show slight differences in their knowledge of board games/card games, other platforms, and especially tablets, show significant deviations from the expected values that can be over ten percentage points in some cases. Some technological platforms such as PC/Mac and hand consoles are preferred at younger ages. On the other hand, other technologies such as board games/card games, tablets, and smartphones are selected by older users. Consoles are the favorite device of middle-aged players between 30 and 35.

Related to game elements, the differences among age intervals seem to be much smaller, not significant for most of the analyzed elements, and “challenges” is one of the game elements that can be remarkable in terms of age differences, decreasing in older adults.

Regarding player preferences on gameful elements in online gaming, the differences among age intervals seem to be much smaller, not significant for most of the analyzed elements. Nevertheless, it should be stated how the use of challenges is the only element that results in noticeable deviations regarding age intervals. The younger and the older age ranges correlate positively with the game element, while the rest of the gaps do the contrary.

Future research should focus on the personalization of games, taking into account the player preferences on gameful elements and preferred platforms/devices according to gender and age. In addition, some further work would naturally follow the presented results. Firstly, statistically, center analysis on a more significant population could clarify the appearing difference in more varied types of games. Secondly, focusing more specifically on relevant targets, such as casual games, would provide more profound insights into game personalization to improve user experience.

## 6. Conclusions

In this paper, we have presented an explorative study about player types and their differences and preferences on game elements, particularly those related to online gaming. The player types and game elements analyzed were selected based on a literature review.

Based on the results, we can highlight the following findings:

- Regarding game design elements, most respondents were familiar with leaderboards and teams. Challenges and exploration were also prevalent, whereas participants knew less about voting and gifting.
- Concerning the gender of the participant population, not only are the numbers imbalanced against the number of females, but also the number of the days per week the female participants play is significantly lower than expected from their number. Female participants exhibited a preference for board games/card games and were penalized for the use of consoles. Tablet use seemed to behave neutrally in terms of participants’ gender. Additionally, female participants did not seem to appreciate the game elements, especially in the case of voting mechanisms,
- The age of participants has also affected the usage of games. At younger ages, the intensity of use of games per week is more significant. Additionally, differences in the preferred device/platform were found, and board games/card games were popular among all age intervals. In contrast, other platforms such as tablets and hand consoles showed more significant differences. Finally, it has been shown how the preference for game elements is much less dependent on the participant’s age, except for the appearance of challenges.

The results shown in the paper contribute to a better understanding of players’ relation to games and the possible improvement of the user experience of online games. Despite the direct relations of gender and age with game platforms and elements that have been analyzed, the presented analysis has limitations in the conclusions to some extent. Although some significant differences have been stated, the available information could not allow us to discover the leading causes of those differences. A more focused analysis on hypothesis statistical validation could further find the reasons behind the differences and establish the path to user experience improvement.

## Figures and Tables

**Table 1:** Game elements in casual online games. Source: own elaboration.

Game Elements in Casual Online Games	Description
Leaderboards/Competition	A way to classify and order users’ performance, for example, using one leaderboard or multiple leaderboards (even one for every activity), tracking different aspects of the game so everyone can compare their capability with others.
Challenges/Achievement	Missions that users can accomplish in the game. They provide a purpose and motivation for the player to reach rewards as achievements. Achievements can be easy, challenging, surprising, and motivating; they are often viewed as levels (Angry Birds), points (Pac-Man), etc.
Points/Credits/Rewards	Usually, collected points can be exchanged with rewards and challenges, shown in the user’s status on leaderboards, or used to obtain virtual goods.
Levels/Status	The levels system provides milestones that players have to reach and often can be shared and shown in the user’s status. It can also be points that players gain to level up, granting them access to new content, rewards, items, etc.
Voting	The multiplayer voting element allows players to cooperate and vote on the choices in-game.
Exploration	One of the reasons why people like to play video games is the potential for exploration. Discovery of new levels, new places, new items, and new plots makes players feel more part of the game or the world.

**Table 2:** Yee’s player motivation categories (Source: Yee (2006) [24]).

Achievement	Social	Immersion
Advancement	Socializing	Discovery
Progress, Power	Casual Chat, Helping	Exploration, Lore, Finding
Accumulation, Status	Others, Making Friends	Hidden Things
Mechanics	Relationship	Role-Playing
Numbers, Optimization	Personal, Self-Disclosure	Story Line, Character
Templating, Analysis	Find and Give Support	History, Roles, Fantasy
Competition	Teamwork	Customization
Challenging Others	Collaboration, Groups	Appearances, Accessories
Provocation, Domination	Group Achievements	Style, Color Schemes
		Escapism
		Relax, Escape from Real
		World, Avoid Real World
		Problems

**Table 3:** Gender distribution of the sample.

Gender	Number	Percentage
Female	338	41.47%
Male	470	57.67%
Other	7	0.86%
Total	815	100.00%

**Table 4:** Native language by gender on the sample.

Native Language	Female	Male	Other	Total
Catalan	27	48		75
English	75	67	4	146
Other	34	49	2	85
Portuguese	29	19		48
Spanish	172	286	1	459
Total	337	469	7	813

**Table 5:** Difference of gender in days of play.

	Female (%)	Male (%)	Other (%)	Total	Avg. Female	Avg. Male	Avg. Other
QH1	1036 (37.74%)	1831 (63.16%)	32 (1.10%)	2867 (100%)	3065	3896	4571

QH1 = How many days in a typical week you usually play games for at least 10 min.

**Table 6:** Gender differences regarding the type of device the participants prefer to use to play.

	Female			Male			Other			Total
	#	%	Dif.	#	%	Dif.	#	%	Dif.	
QP1	109	43.95	8.22	136	54.84	−8.32	3	1.21	0.11	248
QP2	118	28.37	−7.37	294	70.67	7.51	4	0.96	−0.14	416
QP3	89	43.00	7.26	117	56.52	−6.64	1	0.48	−0.62	207
QP4	215	41.11	5.37	305	58.32	−4.84	3	0.57	−0.53	523
QP5	22	26.19	−9.55	59	70.24	7.08	3	3.57	2.47	84
QP6	43	20.87	−14.86	160	77.67	14.51	3	1.46	0.35	206

On which of these platforms do you usually play? QP1: board games/card games; QP2: PC/Mac; QP3: tablet; QP4: smartphone; QP5: hand console; QP6: console.

**Table 7:** Gender differences regarding game element preferences.

	Female			Male			Other			Total
	#	%	Dif.	#	%	Dif.	#	%	Dif.	
QE1	179	35.45	−0.29	321	63.56	0.40	5	0.99	−0.11	505
QE2	154	33.62	−2.11	300	65.50	2.34	4	0.87	−0.23	458
QE3	120	30.77	−4.97	265	67.95	4.79	5	1.28	0.18	390
QE4	35	23.49	−12.25	112	75.17	12.01	2	1.34	0.24	149
QE5	101	37.41	1.67	167	61.85	−1.31	2	0.74	−0.36	270
QE6	117	30.79	−4.95	258	67.89	4.74	5	1.32	0.21	380

**Table 8:** Age difference and days of playing games.

	0 < 20	20 < 25	25 < 30	30 < 35	35 < 40	40 < 45	45 < 50	50<
QH1	203	629	619	468	407	257	156	164
%	6.99%	21.67%	21.32%	16.12%	14.02%	8.85%	5.37%	5.65%
Dif.	−2.92%	1.84%	2.35%	−1.01%	1.41%	−0.94%	−0.13%	−0.59%

**Table 9:** Age difference in using different platforms/devices.

	0 < 20	20 < 25	25 < 30	30 < 35	35 < 40	40 < 45	45 < 50	50<
QP1	17	47	55	35	43	27	13	11
QP2	36	104	92	59	51	34	20	21
QP3	1	22	31	42	36	39	16	20
QP4	32	99	122	81	80	57	27	26
QP5	8	26	23	14	6	4	0	3
QP6	15	44	42	49	30	16	7	3

**Table 10:** Age difference in behavior in games.

	0 < 20		20 < 25		25 < 30		30 < 35		35 < 40		40 < 45		45 < 50		50<	
	%	Dif.	%	Dif.	%	Dif.	%	Dif.	%	Dif.	%	Dif.	%	Dif.	%	Dif.
QH1	7.00		21.68		21.34		16.13		14.03		8.86		5.38		5.58	
QP1	6.85	−0.14	18.95	−2.73	22.18	0.84	14.11	−2.02	17.34	3.31	10.89	2.03	5.24	−0.14	4.44	−1.15
QP2	8.63	1.64	24.94	3.26	22.06	0.72	14.15	−1.98	12.23	−1.80	8.15	−0.71	4.80	−0.58	5.04	−0.55
QP3	0.48	−6.51	10.63	−11.05	14.98	−6.36	20.29	4.16	17.31	3.36	18.84	9.98	7.73	2.35	9.66	4.08
QP4	6.11	−0.89	18.89	−2.79	23.28	1.94	15.46	−0.67	15.27	1.24	10.88	2.02	5.15	−0.22	4.96	−0.62
QP5	9.52	2.53	30.95	9.27	27.38	6.04	16.67	0.53	7.14	−6.89	4.76	−4.10	0.00	−5.38	3.57	−2.01
QP6	7.28	0.28	21.36	−0.32	20.39	−0.95	23.79	7.65	14.56	0.53	7.77	−1.09	3.40	−1.98	1.46	−4.13

**Table 11:** Game elements distributed by age.

	0 < 20	20 < 25	25 < 30	30 < 35	35 < 40	40 < 45	45 < 50	50<
QE1	32	106	103	86	72	55	26	26
QE2	34	108	100	73	61	42	19	22
QE3	30	84	77	64	57	39	16	24
QE4	20	41	38	20	16	7	5	3
QE5	22	59	61	49	35	19	12	15
QE6	30	89	83	56	51	36	19	16

**Table 12:** Game elements distributed by age.

	0 < 20		20 < 25		25 < 30		30 < 35		35 < 40		40 < 45		45 < 50		50<	
	%	Dif.	%	Dif.	%	Dif.	%	Dif.	%	Dif.	%	Dif.	%	Dif.	%	Dif.
QE1	6.32	−0.67	20.95	−0.73	20.36	−0.98	17.00	0.86	14.23	0.20	10.87	2.01	5.14	−0.24	5.14	−0.45
QE2	7.41	0.41	23.53	1.85	21.79	0.45	15.90	−0.23	13.29	−0.74	9.15	0.29	4.14	−1.24	4.79	−0.79
QE3	7.67	0.68	21.48	−0.20	19.69	−1.64	16.37	0.24	14.58	0.55	9.97	1.12	4.09	−1.29	6.14	0.55
QE4	13.33	6.34	27.33	5.65	25.33	4.00	13.33	−2.80	10.67	−3.36	4.67	−4.19	3.33	−2.04	2.00	−3.58
QE5	8.09	1.09	21.69	0.01	22.43	1.09	18.01	1.88	12.87	−1.16	6.99	−1.87	4.41	−0.97	5.51	−0.07
QE6	7.89	0.90	23.42	1.74	21.84	0.50	14.74	−1.40	13.42	−0.61	9.47	0.61	5.00	−0.38	4.21	−1.37

## Data Availability

Not applicable.
